# Magnetic Field Analysis of Lorentz Motors Using a Novel Segmented Magnetic Equivalent Circuit Method

**DOI:** 10.3390/s130201664

**Published:** 2013-01-28

**Authors:** Junbing Qian, Xuedong Chen, Han Chen, Lizhan Zeng, Xiaoqing Li

**Affiliations:** State Key Laboratory of Digital Manufacturing Equipment and Technology, Huazhong University of Science and Technology, 1037 Luoyu Road, Wuhan 430074, Hubei, China; E-Mails: qianjunbing0395@hust.edu.cn (J.Q.); hanchen@mail.hust.edu.cn (H.C.); zenglizhan@mail.hust.edu.cn (L.Z.); xqli@mail.hust.edu.cn (X.L.)

**Keywords:** magnetic equivalent circuit, finite element analysis, Lorentz motor, magnetic flux density, quadratic curve

## Abstract

A simple and accurate method based on the magnetic equivalent circuit (MEC) model is proposed in this paper to predict magnetic flux density (MFD) distribution of the air-gap in a Lorentz motor (LM). In conventional MEC methods, the permanent magnet (PM) is treated as one common source and all branches of MEC are coupled together to become a MEC network. In our proposed method, every PM flux source is divided into three sub-sections (the outer, the middle and the inner). Thus, the MEC of LM is divided correspondingly into three independent sub-loops. As the size of the middle sub-MEC is small enough, it can be treated as an ideal MEC and solved accurately. Combining with decoupled analysis of outer and inner MECs, MFD distribution in the air-gap can be approximated by a quadratic curve, and the complex calculation of reluctances in MECs can be avoided. The segmented magnetic equivalent circuit (SMEC) method is used to analyze a LM, and its effectiveness is demonstrated by comparison with FEA, conventional MEC and experimental results.

## 1. Introduction

In recent years, the Lorentz motor (LM) has been applied widely as an actuator to generate forces with direct drive, fast response time, great precision, low noise, low vibration, *etc.* [1,2]. Despite the above superior performance, some properties like the power-to-weight ratio, efficiency, speed range and cost *etc.*, remain to be improved [3,4]. It has been shown in the literature that flux leakage and magnet end flux have substantial effects on the magnetic analysis [5–8], so that an accurate magnetic flux density (MFD) distribution model of the LM, especially including flux leakage and magnet end flux, is critical.

Different methods, including analytical methods, numerical methods, and magnetic equivalent circuit (MEC) methods, have been employed to model MFD distribution [9]. Analytical methods, based on the Maxwell equations, are a powerful tool, but they can hardly model the slot effect and flux leakage [3,4]. Among numerical methods, FEA is used extensively in the design of motors, however it does not specify the functional form of the relationship between the MFD and geometrical parameters of the motor [10]. Furthermore, the computational cost is enormous.

MEC, based on the Kirchhoff's law, has become an efficient magnetic analysis method [3,9]. Advantages such as moderate accuracy, reduced model complexity and low computational cost, make it an effective means in the design of motors [9,11,12].

MEC was originally proposed and developed in [13–16]. A synchronous machine model was presented in [17–20]. In [21] and [22], mesh-based MECs were discussed. In [5–7,23], flux leakage was modeled by different means, which is crucial in the analysis of motors. Techniques to incorporate MECs with finite-element models were proposed in [24–27]. An accurate yet simple method for predicting the flux density distribution and iron losses in linear PMSM was presented in [28]. In [8], leakage flux associated with a brushless permanent magnet motor utilizing a segmented stator core was analyzed.

However, MEC models always treat the permanent magnet (PM) as one common source and all branches of MEC are coupled together to become a large MEC network. If flux leakage and magnetic end are also considered, the complexity of these models has to be increased and the analysis process would become extremely complicated as well.

This paper presents a segmented magnetic equivalent circuit (SMEC) method, which can be used to analyze the magnetic field of the LM with considerably reduced complexity. The quadratic MFD distribution curves based on the sub-MECs are also proposed to analyze air-gap MFD distribution and to predict the relationship between the air-gap MFD and parameters of the LM. This SMEC method and the curve prediction method are validated by comparison with FEA, conventional MEC method and experimental results.

## 2. Structure of the Lorentz Motor

**Figure 1.**
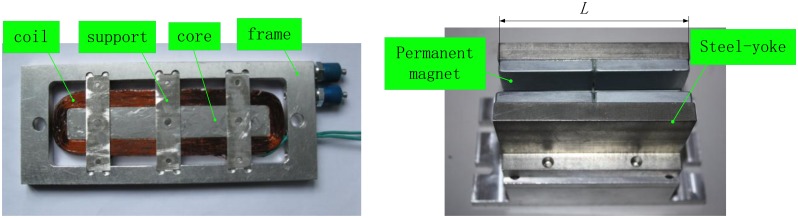
Structure of the Lorentz motor.

A LM is used as the actuator of an isolator since the Lorentz force can be characterized “fast”. Figure 1 shows the structural configuration of the LM. The LM is mainly composed of a mover, a stator and some auxiliaries. The coil is installed in a frame which is sealed by covers.

**Figure 2.**
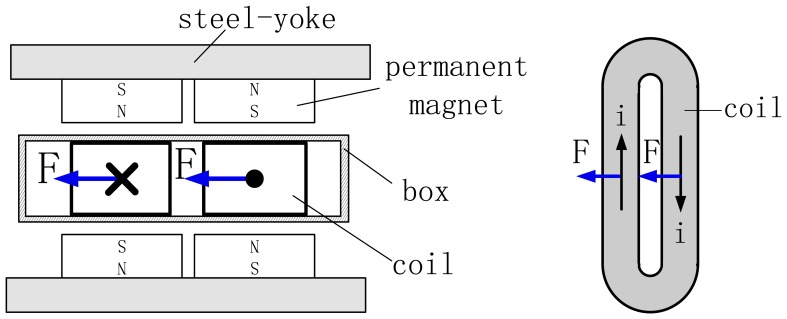
Simplified layout of the Lorentz motor.

The working principle of a Lorentz motor is that a Lorentz force will be exerted on the coil when an electric current flows across it. Electric current I and MFD B are perpendicular to each other, and the direction of the Lorentz force F will be decided by Fleming's left-hand rule. The layout of the LM is illustrated in Figure 2. In order to make the Lorentz force uniform on both sides of the coil, the PMs are stuck on the steel-yoke with alternating polarities. The coil is laid in the air-gap between two opposite magnet poles.

## 3. SMEC Analysis

**Figure 3.**
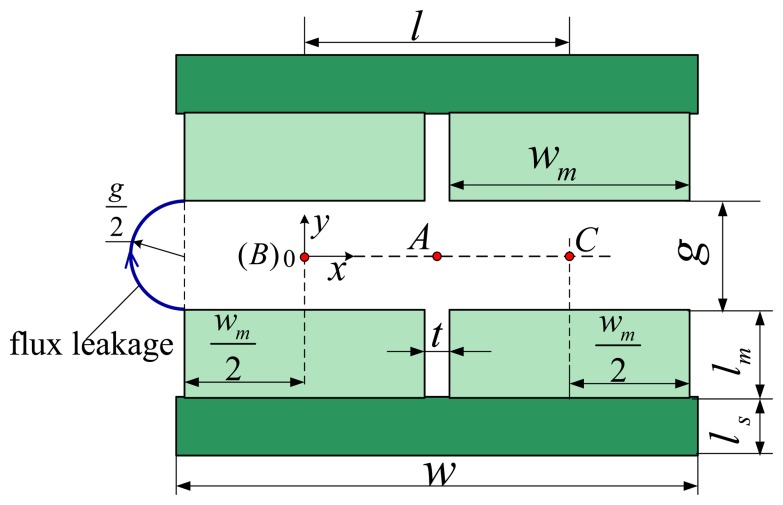
Structure of the stator.

The structure of the stator is shown in Figure 3.

The air-gap between the two magnet poles has width *g; t* is the gap distance between adjacent poles; *l_m_* and *l_s_* represent the thickness of the PM and steel-yoke, respectively. *w* and *w_m_* denote the width of the steel-yoke and PM, respectively.

### 3.1. Conventional MEC

**Figure 4.**
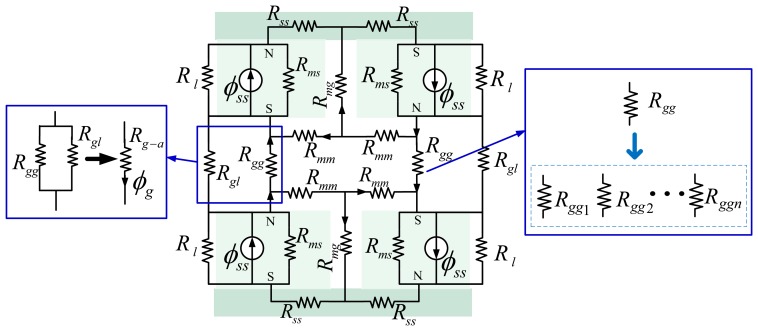
Conventional MEC of the LM stator.

The conventional MEC model [4,17,19,29] of the LM is shown in Figure 4. In Figure 4, *ϕ_ss_* is the flux source of the magnet pole; *R_ms_* stands for the reluctance corresponding to the flux *ϕ_ss_*. *R_gg_* represents the air-gap reluctance. *R_ss_* denotes the reluctance in the steel-yoke. *R_gl_* and *R_l_* are the different leakage reluctances. *R_mg_* denotes the reluctance due to magnet pole-to-pole leakage, and *R_mm_* represents the reluctance of the leakage flux between adjacent poles.

**Figure 5.**
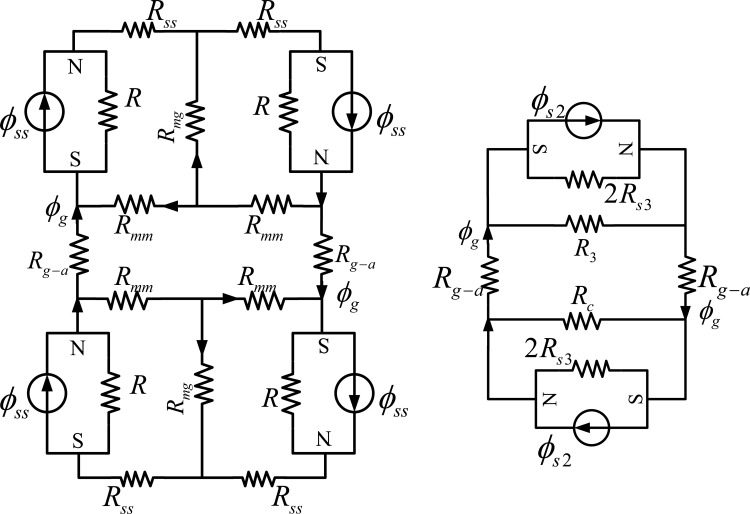
Conventional MEC reduced from Figure 4.

Using the equivalent-resistance theory, the MEC in Figure 4 can be simplified as in Figure 5. The value of these reluctances can be calculated by applying Ampere's law as:

(1) $$R_{gg}=\frac{g}{\mu_{0}w_{m}L},R_{mg}=\frac{l_{m}}{\mu_{0}tL},R_{ms}=\frac{l_{m}}{\mu_{R}\mu_{0}w_{m}L},R_{ss}=\frac{l}{\mu_{s}\mu_{0}l_{s}L}.$$

The reluctance *R_mm_* can be calculated as follows [28]:

(2) $$R_{mm}={\left[\frac{\mu_{0}L}{\pi }\ln(1+\frac{\pi g}{t})\right]}^{-1}$$

The flux leakage tube of *R_gl_* and *R_l_* is a half cylinder and can be expressed as [1,12,29]:

(3) $$R_{gl}=R_{l}=\frac{\pi }{2\mu_{0}L}$$

From Figures 4 and 5 and by flux division, the analytical expressions for reluctance *R_gg_* and *R_gl_* can be obtained as:

(4) $$\phi_{R_{gg}}=\frac{R_{gl}}{R_{gg}+R_{gl}}\phi_{g},\phi_{R_{gl}}=\frac{R_{gg}}{R_{gg}+R_{gl}}\phi_{g},$$

where:

(5) $$\phi_{g}=\frac{\frac{2\frac{R_{1}R_{s2}}{R_{1}+R_{s2}}R_{3}}{2\frac{R_{1}R_{s2}}{R_{1}+R_{s2}}+R_{3}}}{\frac{2\frac{R_{1}R_{s2}}{R_{1}+R_{s2}}R_{3}}{2\frac{R_{1}R_{s2}}{R_{1}+R_{s2}}+R_{3}}}\phi_{s2}$$

and:

(6) $$\phi_{s2}=\frac{\frac{R_{ms}R_{l}}{R_{ms}+R_{1}}}{R_{ss}+\frac{R_{ms}R_{l}}{R_{ms}+R_{1}}}\phi_{ss}$$

(7) $$R_{s2}=(R_{ss}+\frac{R_{ms}R_{l}}{R_{ms}+R_{l}}),R_{1}=2R_{mg}+R_{mm},R_{3}=\frac{2R_{mg}R_{mm}+R_{mm}R_{mm}}{R_{mg}}$$

If the magnetic flux is divided by the corresponding area that the magnetic flux through, the MFD B can be obtained. In the conventional lump-parameter MEC model, the reluctance is modeled by a single constant (e.g., *R_gg_*), thus the spatial variation of MFD cannot be resolved.

In order to model the reluctance more accurately, the air-gap reluctance has to be divided into *R_ggi_* (*I* = 1, 2…*n*) as shown in Figure 4. Similarly, *R_l_*, *R_mg_* and *R_mm_* should also be divided into *R_li_*, *R_mgi_* and *R_mmi_* (*I* = 1, 2…*n*), respectively, resulting in a very large MEC. Since all the circuits are interleaved, the whole MEC has to be solved to obtain any local values. Furthermore, the effect of a local change in the LM will spread over the whole MEC, and the whole MEC has to be solved again to obtain changes on every local values.

### 3.2. MEC Segmentation

In a magnetic field, magnetic flux lines (MFLs) make a closed route from the north to the south and don't cross each other. Similarly, if the MFLs are separated into groups, no group crosses another. In LM, the leakage flux appears at the edge of permanent magnets. If the MEC of the LM can be divided into three sub-MECs and every sub-MEC has independent flux sources and loop, the leakage flux only appears in the lateral groups and the middle MEC is ideal. If the analysis is based on an ideal sub-MEC from all sub-MECs, it will be simple and accurate. For every independent sub-MEC, if parameter of the LM changes in the design, it only affects the corresponding sub-MEC.

**Figure 6.**
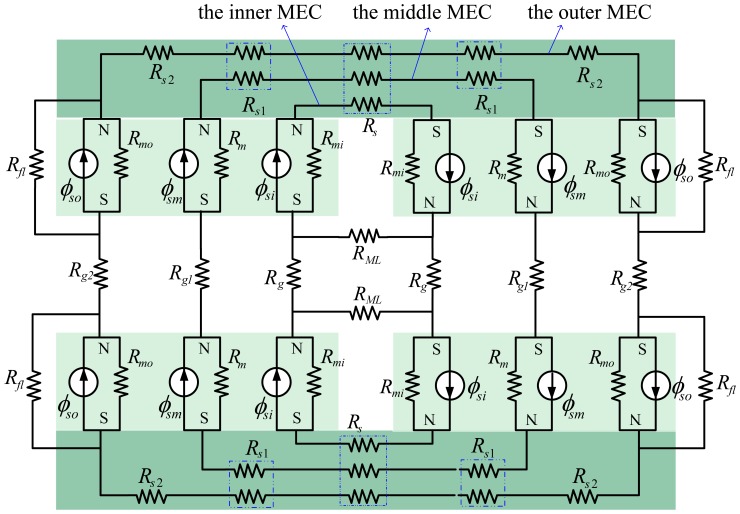
Proposed MEC of the LM stator.

In the design and optimization of motors, it is necessary yet difficult to predict accurately MFD distribution of the air-gap. In order to overcome this problem, quadratic MFD distribution curves based on the analysis of the SMEC are used to obtain the MFD distribution curve of the air-gap. The analysis also holds for nonlinear materials, because the magnetic saturation can be negligible for a LM with larger air gap. Additionally, to avoid magnetic saturation, the geometric size and material properties of the steel-yoke have been chosen elaborately (such that *l_s_* > *l_m_* as shown in Table 1). A 2-D structure of the LM stator and the SMECs are shown in Figures 3 and 6, respectively.

According to the SMEC method, the flux source is divided into three sub-parts (*ϕ_si_*, *ϕ_sm_*, *ϕ_so_*) and three sub-MECs (the inner MEC, the middle MEC and the outer MEC) are sketched in Figure 6.

In all sub-MECs, the middle MEC is the smallest. In its air-gap, MFLs are deemed even without leakage and spreading. The middle MEC is closest to being ideal and affected only slightly by flux leakage in fact. Additionally, the smaller is the middle MEC, the more ideal is this sub-MEC. If necessary, the outer MEC and the inner MEC can be divided further in the segmented decoupling method.

In Figure 6, *ϕ_si_*, *ϕ_sm_* and *ϕ_so_* are the flux sources of the magnet pole in the three sub-MECs, respectively; *R_mi_*, *R_m_* and *R_mo_* stand for the reluctances corresponding to the fluxes *ϕ_si_*, *ϕ_sm_* and *ϕ_so_* respectively. *R_g_*, *R_gl_* and *R_g2_* represent the air-gap reluctances in the three sub-MECs. *R_s_*, *R_s1_* and *R_s2_* denote the different reluctances in the steel-yoke. *R_fl_*is the leakage reluctance of the outer MEC and *R_ML_* is the leakage reluctance of the inner MEC.

### 3.3. Analysis of the Middle MEC

**Figure 7.**
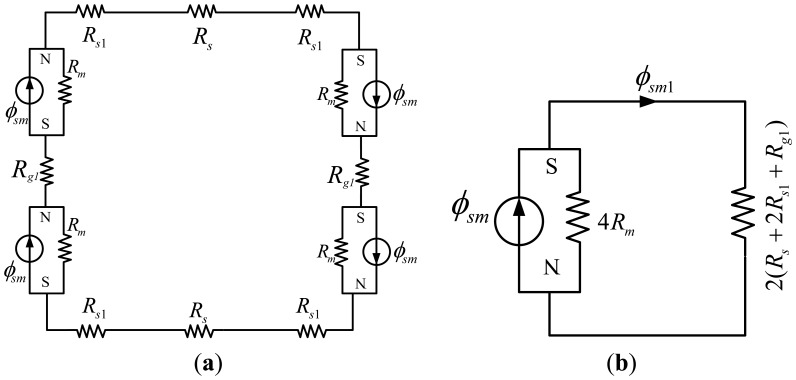
The middle MEC.

Figure 7(a) depicts the middle MEC. It can be simplified as in Figure 7(b), using the equivalent-resistance theory. According to the width of the corresponding flux tubes of *R_s_*, *R_s1_* and *R_s2_* as illustrated in Figure 6, applying the formula of resistance yields:

(8) $$R_{s}=\frac{\frac{w}{5}}{\mu_{s}\mu_{0}\frac{l_{s}}{3}L},R_{s1}=\frac{\frac{w}{5}}{\mu_{s}\mu_{0}\frac{l_{s}}{2}L},R_{g1}=\frac{g}{\mu_{0}A_{g}},R_{m}\frac{l_{m}}{\mu_{rm}\mu_{0}A_{m}}$$

Here, L is the length of the PM in the LM as shown in Figure 1. *A_g_* and *A_m_* represent the flux surface areas of the air-gap and PM in the middle MEC, respectively.

In the middle MEC, *R_s_* + 2*R_s1_* can be expressed as:

(9) $$R_{s12}=2R_{s1}+R_{s}\approx \frac{\frac{7}{5}w}{\mu_{s}\mu_{0}l_{s}L}$$

By flux division:

(10) $$\phi_{g1}=\frac{2R_{m}}{2R_{m}+(R_{s}+2R_{s1}+R_{g1})}\phi_{sm}=\frac{2\frac{l_{m}}{\mu_{0}\mu_{rm}A_{m}}}{2\frac{l_{m}}{\mu_{0}\mu_{rm}A_{m}}+R_{s12}+\frac{g}{\mu_{0}A_{g}}}\phi_{sm}$$

(11) $$B_{g1}=\frac{2\frac{l_{m}}{\mu_{0}\mu_{rm}A_{m}}}{2\frac{l_{m}}{\mu_{0}\mu_{rm}A_{m}}+R_{s12}+\frac{g}{\mu_{0}A_{g}}}B_{sm}$$

Substituting Equation (9) into Equation (11) yields:

(12) $$B_{g1}=\frac{2\frac{l_{m}}{\mu_{rm}A_{m}}B_{sm}}{2\frac{l_{m}}{\mu_{rm}A_{m}}+\frac{\frac{w}{2}}{\mu_{s}\frac{l_{sm}}{w_{m}}l_{s}L}+\frac{g}{A_{g}}}$$

Here, *ϕ_g1_* is the air-gap flux that passes through *R_g1_*. *B_sm_* and *B_g1_* are MFDs corresponding to fluxes *ϕ_sm_* and *ϕ_g1_*, respectively. *A_sm_* is the flux surface area of the magnet pole. *μ_0_* and *μ_rm_* represent air permeability and the relative permeability of the PM, respectively. *H_c_* denotes the magnetic coercive force.

### 3.4. Analysis of the Inner MEC

**Figure 8.**
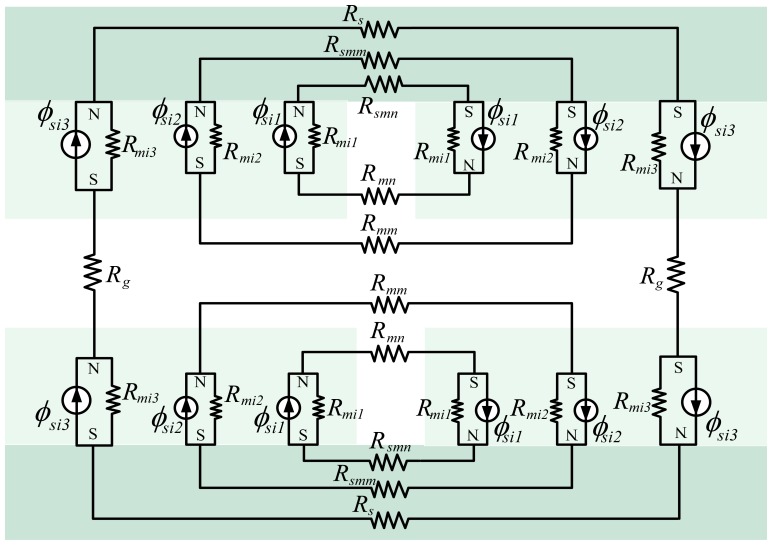
The detailed inner MEC.

The inner MEC can be divided further with the above method as illustrated in Figure 8. The inner MEC is composed of three sub-MECs as shown in Figure 8.

By definition, *ϕ_si_*_1_ and *ϕ_si2_* are the flux-leakage sources of the magnet pole in gap t and air-gap *g*, respectively. *ϕ_si3_* is the flux source of the magnet pole; *R_mi1_*, *R_mi2_* and *R_mi3_* are the reluctances corresponding to fluxes *ϕ_si_*_1_, *ϕ_si2_* and *ϕ_si3_*, respectively. *R_mn_* and *R_mm_* represent the different reluctances due to magnet pole-to-pole leakage. *R_smn_*, *R_smm_* and *R_s_* denote the reluctances of the steel-yoke in different MECs.

**Figure 9.**
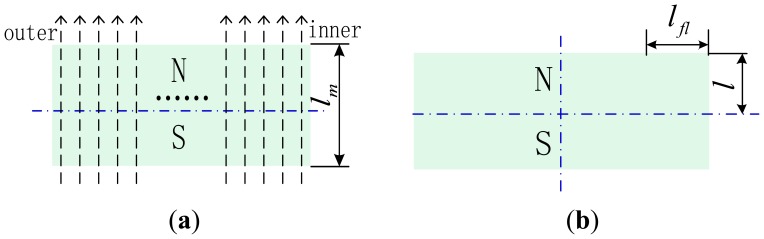
PM. (**a**) ideal MFL of the PM. (**b**) size of the PM.

Here, it is assumed that under ideal conditions, MFLs pass through the PM evenly without leakage as illustrated in Figure 9(a). Then, magnet-end pole-to-pole leakage occurs. Here, *l_m_* is the thickness of the magnet pole and *l_fl_* is the width of the flux source of flux leakage as shown in Figure 9(b). Because flux leakage occurs mainly near the ends of the magnet pole, the width of the flux source of flux leakage *l_fl_* must be smaller than half of *l_m_*. In addition, for weak flux leakage, *l_fl_* is assumed to satisfy *lm*/4 = 1.875 mm.

A reference frame, *x-o-y*, is attached on the central point of the air-gap as illustrated in Figure 3. On the symmetry line of air-gap g, the MFD decreases gradually. It is further assumed that MFD *B_y-in_* can be expressed as a function of *x*. For simplicity, it is also assumed that this function can be written as:

(13) $$B_{y-in}=ax^{2}+bx+c$$

Here, *x* is the coordinate in the x-axis, *B_y-in_* represents the MFD at location *x* of y-coordinate axe in Figure 3, and parameters *a*, *b* and *c* are constant. An analytical expression for the above analysis can be written as:

(14) $$\int_{0}^{\frac{w_{m}-2+t}{2}}\left(\alpha x^{2}+bx+c)\text{Ldx}=B_{g1}L\times [(\frac{w_{m}}{2}-1)-l_{fl}\right]$$

**Figure 10.**
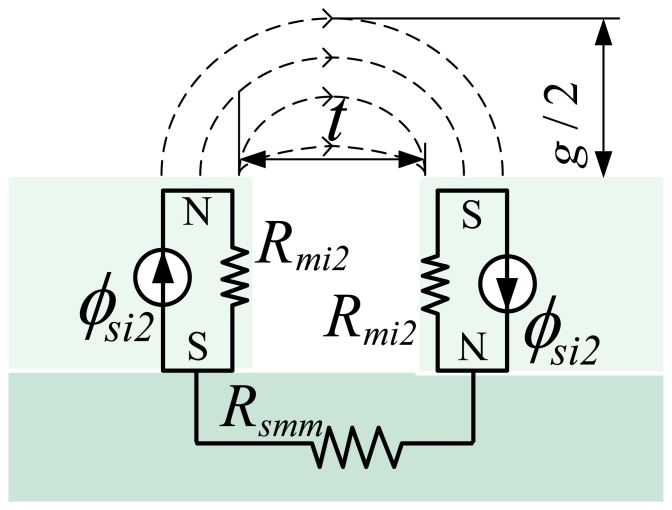
The flux leakage model of the inner MEC.

The route of the flux leakage can be regarded approximately as an ellipse as shown in Figure 10. Since the MFD of air-gap g is symmetrical about the x-axis, it can be deduced that the MFD of point-A ((*w_m_+ t*)/2, 0) is 0 as shown in Figure 3 and the analytical expression can be written as:

(15) $$ax^{2}+bx+c{|}_{x=\frac{w_{m}+t}{2}}=0$$

The MFD of the coordinate origin, as shown in Figure 3, can be calculated from:

(16) $$ax^{2}+bx+c{|}_{x=0}=B_{g1}$$

### 3.5. Analysis of the Outer MEC

**Figure 11.**
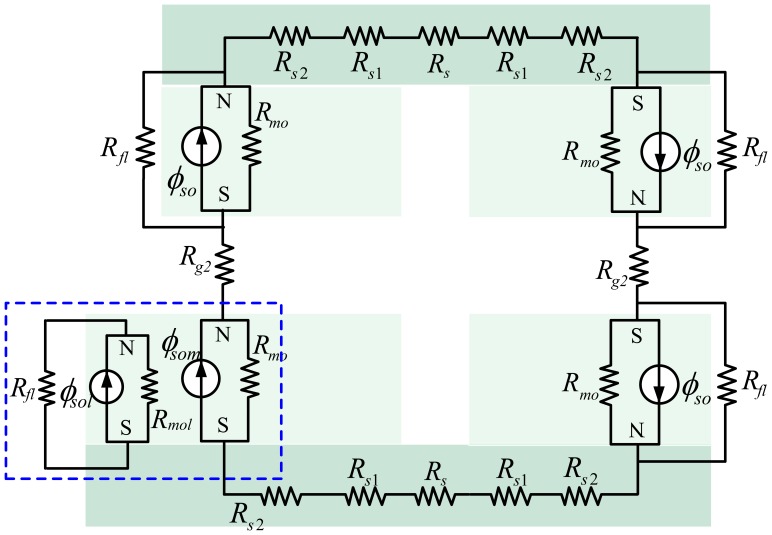
The detailed outer MEC.

Similar to the inner MEC, the outer MEC can be divided further. Since the four sub-MECs are all similar, only one of them (in the dashed box) is divided further as in Figure 11.

Referring to the literatures [1,12,29], the flux leakage tube is a half cylinder as shown in Figure 3. MFD *B_y-out_* can be expressed as a function of *x*:

(17) $$B_{y-\text{out}}=a_{1}x^{2}+b_{1}x+c_{1}$$

Here, *B_y-out_* represents the MFD at location *x* of y-coordinate axe in Figure 3, and parameters *a_1_*, *b_1_* and *c_1_* are constant.

On the boundary of the outer MEC, the MFD can be obtained by solving:

(18) $$a_{1}x^{2}+b_{1}x+c_{1}{|}_{x=-\frac{w_{m}-2+g}{2}}=0$$

(19) $$a_{1}x^{2}+b_{1}x+c_{1}{|}_{x=0}=B_{g1}=0.67$$

When flux leakage is weak, it can be ignored, or simplified as in the analysis of the inner MEC. Since the number of MFL is a constant, the following equation can be obtained:

(20) $$\int_{-\frac{w_{m}-2+g}{2}}^{0}(\alpha_{1}x^{2}+b_{1}x+c_{1})\text{Ldx}=B_{g1}L\frac{w_{m}-2}{2}$$

By dividing every sub-MEC, the whole magnetic field of the LM can be divided into some separate and simple sub-MECs. In this way, every magnetic field of the LM can be analyzed independently and easily.

## 4. Validation of SMEC

2-D FEA has been carried out to validate the SMEC method. The main parameters of the LM are given in Table 1.

**Figure 12.**
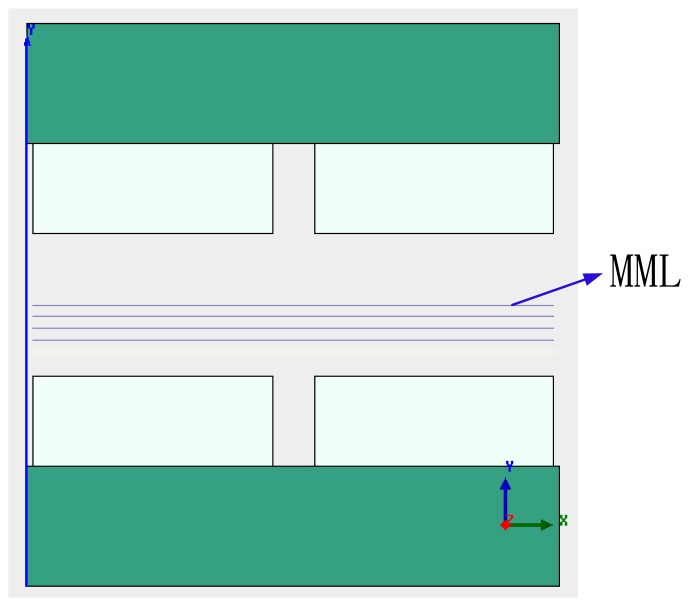
Illustration of the group of lines in the air-gap.

A group of lines that are parallel with the x axis and equally spaced with a distance 1 mm are drawn in air-gap g as shown in Figure 12. In the group of lines, the line of symmetry is called the magnetic middle line (MML) as illustrated in Figure 12.

**Figure 13.**
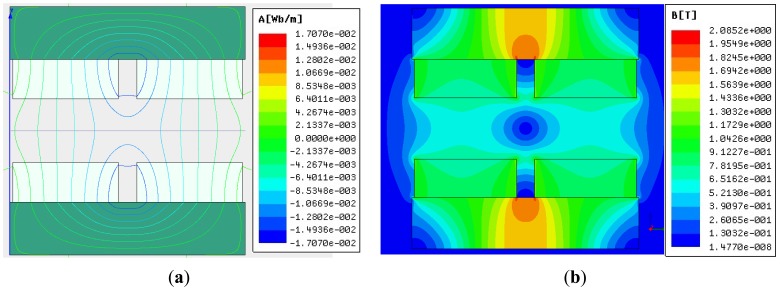
FEA results. (**a**) flux contour profile. (**b**) distribution of magnetic flux density.

Figure 13 shows the flux contour profile and MFD distribution of the air-gap in the LM. Figure 13(b) shows the MFD of the air-gap in the LM. The MFD of point *A* (as shown in Figure 3) is *0*. In the location of the middle sub-MEC, MFLs are nearly ideal.

**Figure 14.**
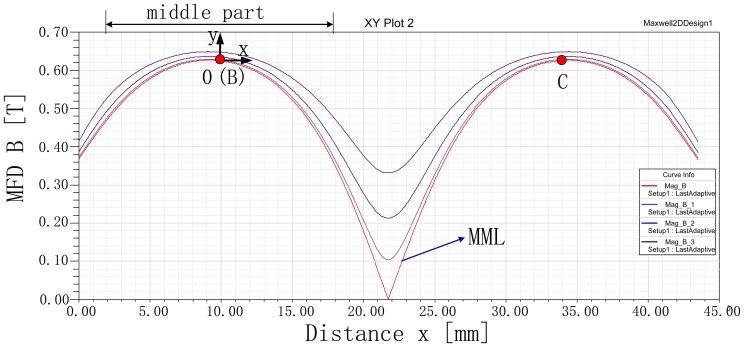
Variation of the MFD with *x*.

Variation of the group of lines with coordinate x is plotted in Figure 14. The curves indicate that the MFD in the middle part of these lines changes less than 0.1 T. The Lorentz force is exerted on the coil placed in this part. Thus, it is critical to analyze the MFD in this middle part. From Figure 14, it can be seen that all the curves have the same qualitative trend.

**Figure 15.**
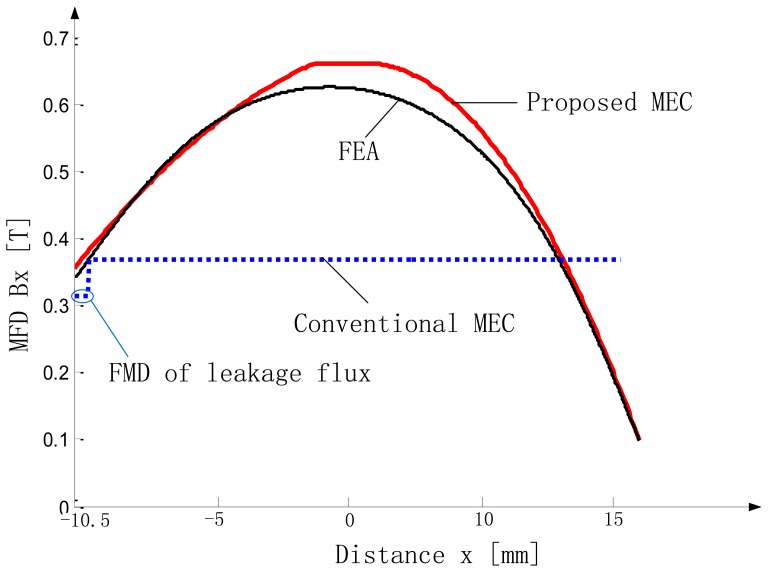
Plot of *B_g1_versus x* with *g* = 12 mm, *t* = 3.5 mm.

The MFD on MML is depicted in Figure 14. Since the curve is symmetric, it is enough to study half of it. In Figure 15, the curves, obtained from the proposed SMEC (Equations (5)–(7) and (9)–(11)), 2-D FEA and conventional MEC, are shown for comparison.

As illustrated in the figure, results of the proposed SMEC method are in very good agreement with 2-D FEA and the difference is less than 6%, while the difference between conventional MEC analysis and 2-D FEA is much larger.

**Figure 16.**
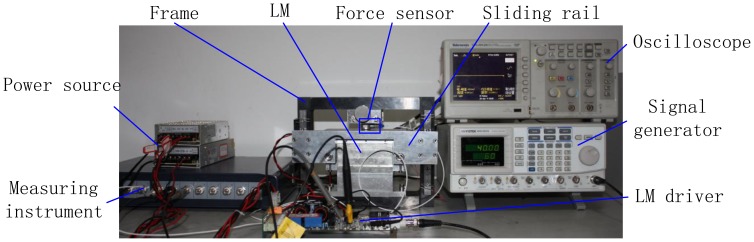
Photo of experimental setup.

**Figure 17.**
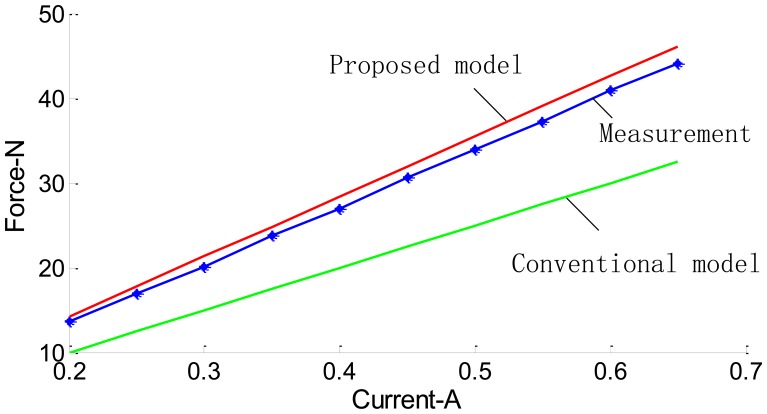
Plot of F *versus* I with n = 636.

To obtain the relation between thrust force and current in the LM, the corresponding experiment is conducted as shown in Figure 16. After the air-gap MFD of the LM has been obtained, according to the Lorentz law, the thrust force of the LM is calculated using the integral method [1,30]. Figure 17 shows the thrust as a function of primary current density. It can be seen that the proposed SMEC method is more accurate than the existing method.

From Figures 15 and 17, it is evident that the spatial variation of the air-gap MFD can be obtained more accurately by the SMEC method than the conventional MEC method. Thus, the thrust of the LM can be predicted more accurately. This SMEC method can be very effective in the design and optimization of LMs.

In the proposed SMEC method (Figure 6), each sub-MEC can be solved independently, and any local change will affect only the local sub-MEC. In other words, using the SMEC method, modeling of the LM can be parallelized, and the computational gain increases significantly with the increase of the number of elements in the LM. At the same time, with our proposed SMEC method, spatial variation of MFD can be resolved accurately (Figure 15), which is another advantage over the conventional MEC method.

## 5. Conclusions

A simple yet accurate SMEC method for predicting air-gap MFD distribution of LMs is proposed, in which segmented sub-MECs are decoupled. The magnetic field of the LM can be analyzed with considerably reduced complexity and the relation between the air-gap MFD and the parameters of LM can be established easily. The size of middle sub-MEC is the smallest one, which approaches an ideal situation and can be solved accurately by MEC equations. In the middle part of the LM air-gap, the MFD is approximately uniform. Based on the study of the middle MEC, relationship between this part of the MFD and parameters of the LM can be obtained by analyzing the middle MEC. After analyzing sub-MECs, quadratic curves are used to predict the air-gap MFD of the LM. The calculation of complex reluctances of MECs is avoided. Prediction accuracy of the proposed method is verified by comparison with FEA results, and is less than 6%. The comparison between proposed SMEC and conventional MEC shows the advantage of the proposed SMEC. The proposed SMEC method can be used in LM design and optimization with improved simplicity and desirable accuracy.

## Figures and Tables

**Table 1.** Parameters of the LM.

**Parameter**	**Value**	**Unit**
*μ_0_*	4π × 10^−7^	H/m
*μ_rm_*	1.02	
*μ_s_*	400	
*w_m_*	20	mm
*l_m_*	7.5	mm
*l_s_*	10	mm
*L*	100	mm
